# Enzyme-Triggered Intestine-Specific Targeting Adhesive Platform for Universal Oral Drug Delivery

**DOI:** 10.1002/adhm.202301033

**Published:** 2023-06-30

**Authors:** Ying Li, Jung Seung Lee, Ameya R. Kirtane, Mengyuan Li, Charles William Coffey, Kaitlyn Hess, Aaron Lopes, Joy Collins, Siddartha Tamang, Keiko Ishida, Alison Hayward, Jacob Wainer, Adam J. Wentworth, Giovanni Traverso

**Affiliations:** Institute of Medicinal Plant Development, Chinese Academy of Medical Sciences and Peking Union Medical College, Haidian District, Beijing 100193, P. R. China; David H. Koch Institute for Integrative Cancer Research, Massachusetts Institute of Technology, Cambridge, MA 02139, USA; Department of Intelligent Precision Healthcare Convergence, Sungkyunkwan University, Suwon 16419, South Korea; Department of Biomedical Engineering, Sungkyunkwan University, Suwon 16419, South Korea; David H. Koch Institute for Integrative Cancer Research, Massachusetts Institute of Technology, Cambridge, MA 02139, USA; Division of Gastroenterology, Brigham and Women’s Hospital, Harvard Medical School, Boston, MA 02115, USA; David H. Koch Institute for Integrative Cancer Research, Massachusetts Institute of Technology, Cambridge, MA 02139, USA; Faculty of Applied Science & Engineering, University of Toronto, Toronto, ON M5S1A4, Canada; Department of Biological Engineering, Massachusetts Institute of Technology, Cambridge, MA 02139, USA; David H. Koch Institute for Integrative Cancer Research, Massachusetts Institute of Technology, Cambridge, MA 02139, USA; David H. Koch Institute for Integrative Cancer Research, Massachusetts Institute of Technology, Cambridge, MA 02139, USA; David H. Koch Institute for Integrative Cancer Research, Massachusetts Institute of Technology, Cambridge, MA 02139, USA; David H. Koch Institute for Integrative Cancer Research, Massachusetts Institute of Technology, Cambridge, MA 02139, USA; David H. Koch Institute for Integrative Cancer Research, Massachusetts Institute of Technology, Cambridge, MA 02139, USA; David H. Koch Institute for Integrative Cancer Research, Massachusetts Institute of Technology, Cambridge, MA 02139, USA; Division of Comparative Medicine, Massachusetts Institute of Technology, Cambridge, MA 02139, USA; David H. Koch Institute for Integrative Cancer Research, Massachusetts Institute of Technology, Cambridge, MA 02139, USA; David H. Koch Institute for Integrative Cancer Research, Massachusetts Institute of Technology, Cambridge, MA 02139, USA; Department of Mechanical Engineering, Massachusetts Institute of Technology, Cambridge, MA 02139, USA; David H. Koch Institute for Integrative Cancer Research, Massachusetts Institute of Technology, Cambridge, MA 02139, USA; Division of Gastroenterology, Brigham and Women’s Hospital, Harvard Medical School, Boston, MA 02115, USA; Department of Mechanical Engineering, Massachusetts Institute of Technology, Cambridge, MA 02139, USA

**Keywords:** enzyme-triggered adhesives, intestinal targeting, mucoadhesives, mussel inspired adhesives, oral drug delivery systems

## Abstract

Patient adherence to chronic therapies can be suboptimal, leading to poor therapeutic outcomes. Dosage forms that enable reduction in dosing frequency stand to improve patient adherence. Variation in gastrointestinal transit time, inter-individual differences in gastrointestinal physiology and differences in physicochemical properties of drugs represent challenges to the development of such systems. To this end, a small intestine-targeted drug delivery system is developed, where prolonged gastrointestinal retention and sustained release are achieved through tissue adhesion of drug pills mediated by an essential intestinal enzyme catalase. Here proof-of-concept pharmacokinetics is demonstrated in the swine model for two drugs, hydrophilic amoxicillin and hydrophobic levodopa. It is anticipated that this system can be applicable for many drugs with a diverse of physicochemical characteristics.

## 1. Introduction

The Centers for Disease Control (CDC) estimates that 20–30% of new prescriptions are never filled at the pharmacy and medications are not taken as prescribed 50% of the time. Importantly, patient nonadherence causes 30–50% of chronic disease treatment failures^.[1]^ The number of pills per dose and the number of doses per day are key factors that contribute to patient nonadherence.^[2]^ Short elimination half-time of drugs causes a high pill burden, especially from long-term medication.

Two therapeutic areas that stand to benefit significantly from improved patient adherence are infectious diseases and Parkinson’s disease (PD). Poor adherence to anti-infective therapy may lead to therapeutic failure, re-infection, and resistance. For example, in a study analyzing treatment outcomes of patients treated with suspected bacterial infections of the lower respiratory tract, pharyngitis and dental infections, patients in the twice-daily groups had a higher bacteriologic failure rate in comparison to the once-daily group (7.1% vs 2.8% for the once-daily group).^[3–5]^ PD resulted in 3.2 million (95% uncertainty interval (UI) 2.6–4.0) disability-adjusted life-years (DALYs) and 211296 deaths (95% UI 167771–265160) in 2016.^[6]^ More than one third of PD patients, who despite taking three or more daily drug doses, experience severe motor fluctuations, fatigue, cognitive impairment and/or mood disturbances. This leads to reported low adherence to their pharmacotherapy.^[7,8]^ Extended-release carbidopa-levodopa showed significantly longer duration than immediate-release carbidopa-levodopa (mean duration of effect: 5.56 vs 2.69 h), followed with a ≥20% improvement in finger-tapping, a ≥11-point improvement in Unified Parkinson’s Disease Rating Scale (UP-DRS) motor score.^[9]^ To solve these problems and increase patient adherence, there is a critical need to develop orally-delivered sustained release platforms that can reduce dosing frequency and simplify drug administration.

Our group has previously developed gastric resident drug delivery systems and shown their utility for the delivery of anti-infective and contraceptive drugs.^[10–15]^ However, given these systems are limited by the volume that is encapsulated in an ingestible capsule drugs which require significant daily dosages such as antibiotics and drugs for the treatment of PD may not be amenable to being delivered using these systems.

Mucoadhesives have served as a key element of orally administered drug-delivery systems as they help to prolong gastrointestinal (GI) residence time and provide controlled drug release at a targeted region,^[16,17]^ which could help reducing the frequency of drug administration.^[18–22]^ Oral mucoadhesive drug delivery systems are generally noninvasive, thereby avoiding the uncomfortable aspects of intravenous, intramuscular, or subcutaneous delivery methods. Encapsulation of the drug in mucoadhesive systems may enable longer residence at the site of absorption and improved bioavailability.^[23]^ However, mucoadhesive polymers are usually macromolecular, hydrophilic gelling substances with numerous hydrogen-bond forming groups. These polymers adhere to tissues through nonspecific, noncovalent interactions that are primarily electrostatic in nature, such as hydrogen bond, ionic interactions.^[24,25]^ Here, the present mucoadhesive materials have no specific bio-adhesion properties to intestine and show only weak adhesion in the hydrated environment of body.^[26–29]^ The current strategy that promote intestine-specific targeting adhesion through covalent binding includes catalyst-free thiol-disulfide exchange between mucin associated disulfides and newly converted thiols on bacterial surface to develop gut microbiota as oral therapeutics targeted treatment of colitis.^[30]^ Incorporation of drug into other biological interfaces by dopamine have been explored to enhance biological action,^[31–35]^ but dopamine’s robust adhesion and it’s intestine-specific adhesive system weren’t explored.

We developed an enzyme-triggered small intestinal targeting adhesion technology termed gastrointestinal synthetic epithelial lining (GSEL).^[36]^ GSEL enables strong tissue adhesion through intestinal catalase (CAT) catalyzed polymerization of dopamine (DA) to poly(dopamine) (PDA). The hydroxyl groups of DA can be converted into ortho-quinone (o-quinone) moieties that can react via Michael type addition or Schiff base reaction with nucleophilic groups (NH_2_, SH, OH, COOH) exposed on different types of tissue surfaces, thus leading to the formation of covalent bonds.^[37,38]^ In order to further enhance the capabilities of this system we sought integrate some of the fundamental advantages of hydrogels together with DA.

Therefore, based on the special mechanism for specific targeting and localization to the small intestine of our developed GSEL system, we first synthesized catechol-functionalized polymer, modifying alginate (ALG) backbones with DA (ALG-DA). Mixing the ALG-DA with other GSEL ingredients can enable gelation on the surface of the small intestine due to the presence of the enzyme – CAT to form the 3D hydrogel system. The oxidized catechol groups of DA can covalently react with biomolecules on the small intestine, making a robust adhesion in the hydrated intestinal environment. Then, we designed a multi-layered pill from the ALG-DA mixture. This consisted of two outside layers capable of forming an adhesive hydrogel with a drug reservoir between the adhesive layers. The drug reservoir layer is capable of supporting hydrophilic and hydrophobic drugs. We further demonstrated proof-of-concept intestine-specific resident drug delivery system in a large animal model. We believe our platform has the ability to load and release drugs in a controlled and sustained manner, and can be applied toward a broad set of active pharmaceutical ingredients.

## 2 Results

### 2.1. Design of an Oral Pill Capable of Prolonging Intestinal Residence

We set out to design an oral dosage form that has the ability to specifically adhere to the small intestine, sufficiently load various kinds of the therapeutic agents, provide controlled release of the agents and show good biocompatibility.^[39]^

**Figure 1.**
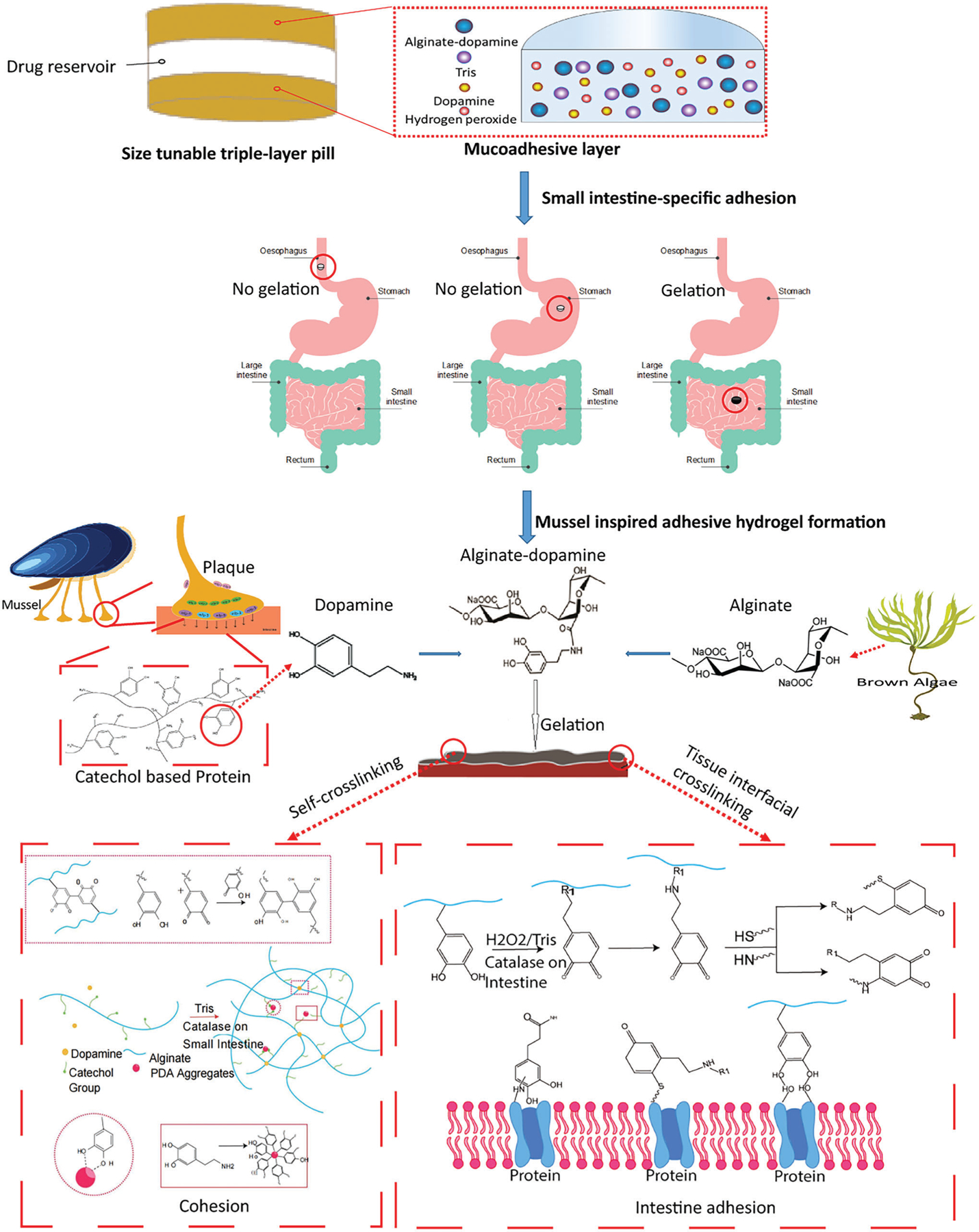
Schematic depicting of tissue-adhesion dosage form. Schematic illustration of sandwiched triple-layer pills, and gelation and adhesion process of Alginate-dopamine on the small intestine inspired by enzyme.

In order to load different drugs and not affect the adhesive property, we designed a modular system where a sustained release matrix of drug was located between two layers of mucoadhesive polymer. Most mucoadhesive polymers rely on weak non-specific interactions for adhering to the wall of the gastrointestinal tract. We hypothesized that covalent interactions targeted to specific regions of the gastrointestinal tract could lead to longer retention and more reproducible results. To achieve this, we used a dopamine-conjugated ALG polymer in the mucoadhesive layer. ALG has been widely applied as a drug carrier due to its biocompatibility, biodegradability, and relatively low cost.^[40,41]^ Further the mucoadhesive layer contained Tris base to provide buffering capacity and hydrogen peroxide as a source of oxygen. In the presence of intestinal CAT,^[42]^ hydrogen peroxide is converted to oxygen which leads to the oxidation of the dopamine side chains on ALG. This in turn leads to self-cross linking through Michael addition and Schiff base reaction to form hydrogel.^[43]^ The nanoparticles (PDA) from GSEL ingredients can coordinate with ALG-DA hydrogel to enhance the cohesion of hydrogel. Importantly, oxidized ALG-DA covalently reacts with nucleophilic amine or thiol groups present in the tissue. The coordination and covalent bonds endow robust mucoadhesion in the hydrated intestinal environment. A schematic depicting our modular sustained release drug delivery system and the strategy for dopamine-based in situ gelation and adhesion on intestine were shown in Figure 1.

### 2.2. Alginate-Dopamine Preparation and Characterization

**Figure 2.**
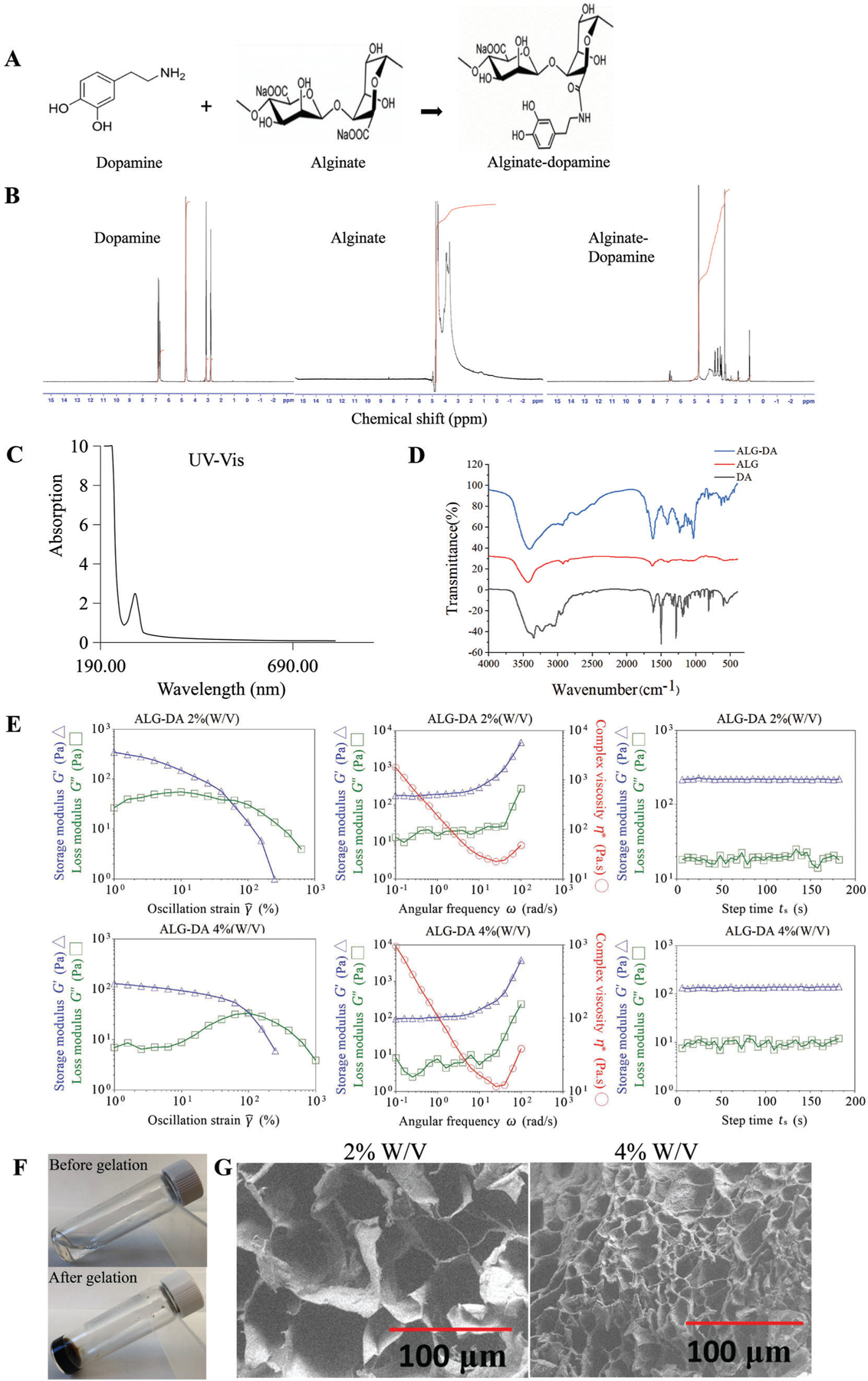
Characterization of alginate-dopamine. A) The synthesis process of alginate-dopamine. B) 1H NMR spectra of alginate, dopamine and alginatedopamine. C) UV–Vis spectra of alginate-dopamine. D) FTIR spectra of alginate, dopamine and alginate-dopamine. E) Rheological properties of the alginate-dopamine hydrogels. The storage (G′) and loss (G″) moduli of the 2% (w/v) alginate-dopamine hydrogel and 4% (w/v) alginate-dopamine hydrogel at a frequency sweep mode are provided. F) The gelation photos of ALG-DA. G) SEM images of alginate-dopamine hydrogel. Scale bar, 100 μm.

The ALG-DA was synthesized by standard carbodiimide coupling chemistry (EDC/NHS chemistry). In this reaction, the carboxyl group of ALG was activated by EDC/NHS and the amine group of dopamine was then coupled to the activated carboxyl group (Figure 2A). The conjugation efficiency was determined by measuring absorbance at 280 nm using a UV−Vis spectrophotometer. We found that the conjugation of DA to polymer backbone depends on the molar ratio of ALG: DA. Specifically, the conjugation efficiency of DA to ALG backbone was 11.77 ± 2.64% and 15.69 ± 1.43% at ALG: DA molar ratios of 1:1 and 1:2, respectively. We confirmed the conjugation of DA to ALG by H^1^-nuclear magnetic resonance (H^1^-NMR) based peaks of the catechol protons observed at ≈7 ppm (Figure 2B). UV–Vis analysis demonstrated there is characteristic absorption peak at ≈270 nm for ALG-DA, which is the absorbance wavelength of DA (Figure 2C). Similar conclusions were obtained by Fourier transform near infrared (FT-IR) analysis of the polymer. Characteristic absorptions of ALG (C-O-C at 1037 cm^−1^, C–O at 1113 cm^−1^) were identified in each group. Importantly, the peaks at 1G22 and 1402 cm^−1^ were assigned to the N−H deformation and C−N stretching vibrations, respectively, which were remarkably strengthened after DA modification (Figure 2D).

We then characterized the rheological properties, micro-structure, and swelling behavior of hydrogels formed from ALG-DA. To form hydrogels, we mixed the ALG-DA with free DA, hydrogen peroxide, Tris base, and CAT for the in vitro evaluation. Generation of oxygen from hydrogen peroxide promoted the oxidation of DA and cross-linking of the ALG-DA. We investigated the effect of ALG-DA concentration on the rheological behavior of ALG-DA hydrogels using a TA Instruments DHR-2 Rheometer. Oscillatory measurements indicate that both the storage modulus (G’) and loss modulus (G’’) of ALG-DA hydrogels increased with concentration. Oscillatory rheological properties were performed to determine the viscoelastic properties of the ALG-DA hydrogels, Samples were subjected to increasing frequency at a strain of 0.01% (Figure 2E). The G’ of the ALG-DA hydrogel was nearly consistent over the frequency range tested and was higher than the G’’ at each frequency in the 2% and 4% ALG-DA hydrogel. The G’ values for the hydrogel were initially independent of oscillation frequency across different concentrations and G’ was greater than the G’’, indicating these hydrogels were chemically cross-linked and that cross-linking in the ALG-DA hydrogel was highly stable and uniform. Higher G’ for hydrogels of higher concentrations likely resulted from a higher degree of cross-linking. Elevated G’’ values indicated strong viscous dissipation properties resulting from the breaking of the hydrogel. At strains ≈1, the G’ values of hydrogels decreased rapidly with the increase of strain, suggesting the gels underwent gel – sol transition and behaved as liquids. The cross point of G’ and G’’, representing the transition of the gel network to a liquid state (solution behavior: G’ < G’’, solid behavior: G’ > G’’) (Figure 2E). Time sweep experiments show that the G’ and G’’ values of ALG-DA hydrogels separately increased from ≈10 and ≈100 Pa to ≈20 and ≈200 Pa with ALG-DA concentration from 2% to 4% (Figure 2E). The precursor solution of ALG-DA formed hydrogel within 30 min of gelation (Figure 2F). Scanning electron microscope (SEM) analysis demonstrated that ALG-DA hydrogel has a well-defined lamellar structure with interconnected pores. The high concentration of ALG-DA hydrogel (4% W/V) produced smaller and more porous specimens than the low concentration of ALG-DA hydrogel (2% W/V) due to increasing of viscosity of the hydrogel solution which prevented the bubbles from escaping from the solution and supported interconnected channels. The relative higher cross-linking agent concentration in low concentration hydrogels resulted in increase in entanglement between monomer and polymer which results in decreased porosity (Figure 2G).

The swelling properties of ALG-DA hydrogel were examined by measuring the change in hydrogel weight during incubation under intestinal physiological conditions (in a PBS solution at 37 °C) (Figure S1, Supporting Information). The swelling ratios of the ALG-DA hydrogels are 192.9 ± 14.5% for 2% (W/V) ALG-DA and 229.7 ± 3.8% for 4% (W/V) ALG-DA. The porosity and gel fraction increased with increase in ALG-DA content, which allows more water to enter the films and support swelling. The swelling behavior of the hydrogel gave an insight into its well-cross-linked network.

Our study demonstrated that catechol-functionalized alginate polymers inspired by mussel adhesive chemistry can generate highly organized and controllable hydrogels.

### 2.3. Gelation In Vitro and In Vivo

Next, we were interested in determining if ALG-DA hydrogels doped with DA could cross-link via polymerization on the surface of the gastrointestinal tract. Additionally, we were interested in understanding the degree of polymerization across the different portions of the gastrointestinal tract.^[44,45]^

The PDA can embed into ALG-DA hydrogel network to strengthen the mechanical strength of hydrogel.^[46]^ The polymerization of dopamine in our hydrogel system was shown in Figure S2 (Supporting Information). After centrifugation to measure the diameter of PDA by dynamic laser scattering (DLS) (Zetasizer Nano ZS90 instrument, Malvern Panalytical), the size of PDA is 469.5 ± 12.20 nm for 0.5% (w/v), 402.26 ± 68.88 nm for 1% (w/v) and 1189.33 ± 54.12 nm for 2% (w/v). When we used 1% DA in our formulations, hydrogels were formed within 20 min. The lower and higher concentration of DA influence the gel formation speed. The lower concentration of DA formed nanoparticles too slowly, while the higher concentration of DA formed much larger aggregates along with a small amount of the desired nanoparticles, which could not efficiently link the polymer together. During oxidation process of DA, some amount of PDA covalently couples on the surface of ALG-DA matrix, which also contributed to the excellent properties of the hydrogel. The lower concentration of DA can’t interact with the DA on the ALG-DA networks enoughly, which leads to the slow gelling. Noting that PDA is a heterogeneous material in which an unpolymerized self-assembled structure is included during oxidative polymerization of DA.^[47]^ The higher concentration of DA forming PDA with large size which is more likely to be a supramolecular aggregate, held together primarily through noncovalent interactions, and does not form long-chain networks.^[48]^

**Figure 3.**
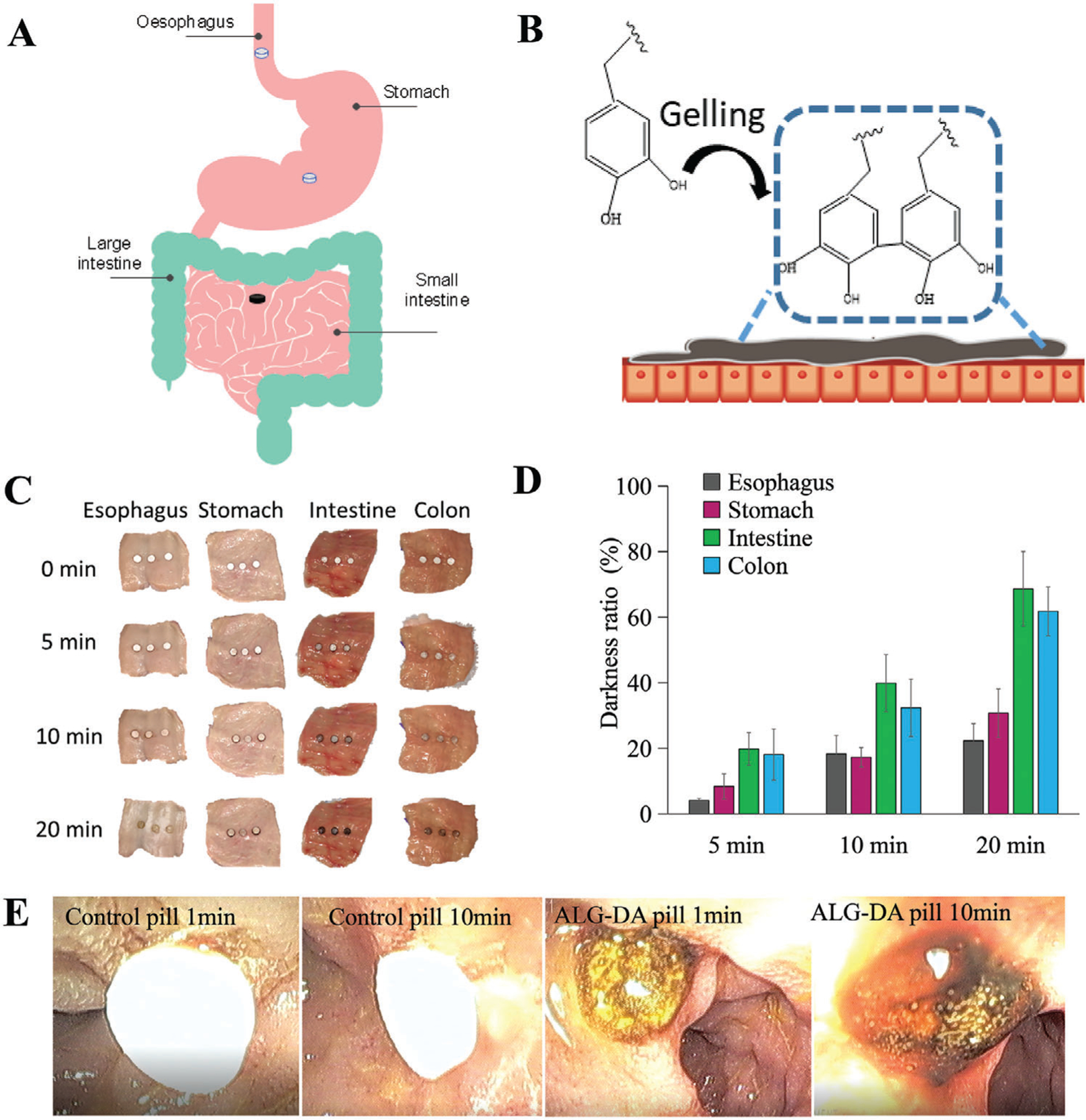
The gelation of alginate-dopamine pills. Scheme of the A) oxidation reaction of alginate-dopamine (ALG-DA) pill on different parts of gastrointestinal (GI) tract B) ALG-DA adhesive hydrogel formation on tissue. C) The photographs of ALG-DA pills gelation on different parts of GI tract of pigs in vitro at different time-points. D) The gelation degree calculation of ALG-DA pills on different parts of GI tract of pigs in vitro. Data is represented as mean ± S.D., *n* = 3, *indicates *p* < 0.05. E) Endoscopic view of ALG-DA pills gelation in vivo after 10 min attachment to small intestine of pigs in vivo.

We placed pills made from ALG-DA, dopamine, hydrogen peroxide, and Tris on the surface of various segments of the porcine GI tract. After cross-linking, the hydrogel was observed to have a brown color due to the oxidation of the catechol moiety followed by polymerization, which is also a sign of gelation (Figure 3A,B). This allowed us to study the degree of polymerization using colorimetric analysis. The process of hydrogel formation on different porcine GI tissues were recorded using digital camera (Figure 3C) and darkness measurements using image J software (Figure 3D). Gels were formed faster on the small intestine compared to the esophagus and stomach, which indicated the potential to target the pills to adhere to the lower GI tract. Gelation of ALG-DA can be modulated by changing the concentration of H_2_O_2_ and Tris base (pH). ALG-DA in our optimized composition ratio of H_2_O_2_ and Tris base (pH 8.5) (ALG-DA: Tris base: H_2_O_2_ = 1:1.21:0.09) could form the tough hydrogel within 20 min. If we decreased the concentration of H_2_O_2_ or increased the concentration of Tris base, the hydrogel formation time increased or failed to complete cross-linking reaction. Basic conditions (pH 8.5) are required to promote and accelerate the redox reaction without strong oxidants in the presence of oxygen.^[49]^ Above pH 8.5, the activity of CAT decreases slowly.^[50]^ Low H_2_O_2_ did not produce sufficient O_2_ to catalyze fast polymerization. Conversely, high H_2_O_2_ can result in reactive oxygen species (ROS) that are associated with various acute and chronic inflammatory diseases. Additionally, an excess of O_2_ impeded the formation of a well-cross-linked network with pores (see Figure S3, Supporting Information for SEM images of hydrogel with different concentrations of H_2_O_2_). Our GSEL ingredients mixture could form a mucoadhesive hydrogel quickly in the presence or upon exposure to CAT. If the ALG-DA is fully oxidized, then the system did not demonstrate tissue adhesion (Figure S4, Supporting Information). When hydrogel-containing pills were delivered to the small intestine, mucoadhesive hydrogel was observed within 10 min (Figure 3E).

In sum, our studies showed that ALG-DA could rapidly polymerize on the surface of the small intestine. The rate of polymerization was influenced by the concentration of pH modulating agents and the oxidizing agent.

### 2.4. Adhesion and Erosion Test In Vitro

**Figure 4.**
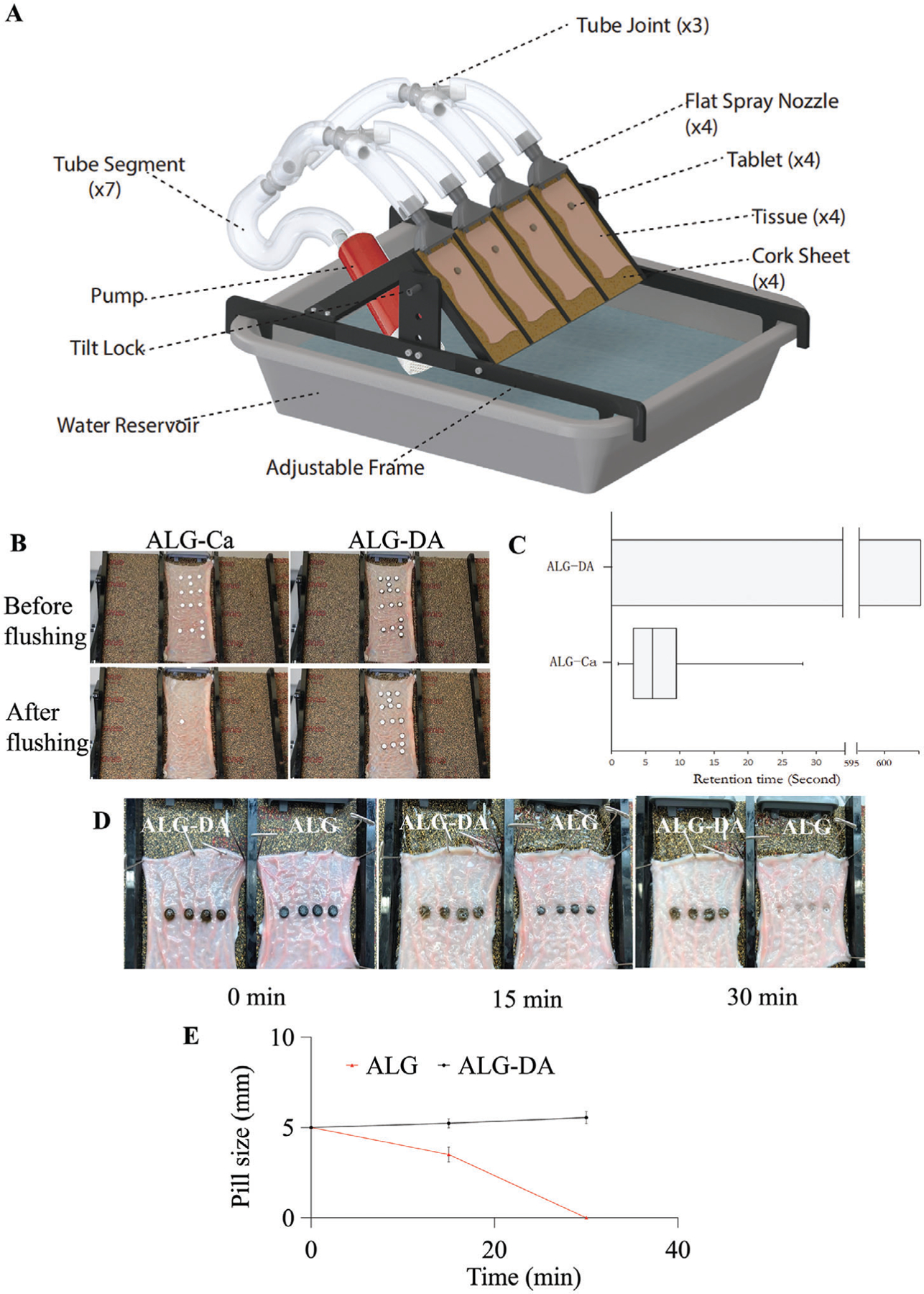
Adhesion and erosion test of alginate-dopamine pills. A) Schematic representation of the experimental washing setup. B) Photographs of alginate-dopamine pills (ALG-DA) and alginate-calcium pills (ALG-Ca) adhesion performance after water flushing within 10 s. C) Retention time of ALG-DA pills and ALG-Ca pills on small intestine after water flushing, *n* = 30. D) Photographs of the erosion of ALG-DA pills and alginate (ALG) pills on small intestine over time. Graphite is added in the ALG pills in order to investigate the erosion process (dark color). E) The pills sizes change ALG-DA pills and ALG pills after water flushing for 30 min. Data is represented as mean ± S.D., *n* = 4.

To test the adhesion of the pills to the surface of the intestine, we developed an adjustable tilted fixture. The setup allows up to 4 flow paths with adjustable angles (15, 30, 45 °); flow can be diverted to adjust volumetric flow through each individual nozzle (Figure 4A). We placed tablets made from alginate, alginate cross-linked with calcium or ALG-DA hydrogels on top of the tissue. Following 10 min incubation time, we flowed water and measured the retention of the pills. ALG-DA pills were retained on the intestine for a longer period in comparison to ALG-Ca pills (Figure 4B). Video for evaluation of dislodgment was recorded with a digital camera (Movies S1 and S2, Supporting Information). Further, we tested the retention time of 30 pills on small intestinal tissue mounted on the flow table, ALG-Ca pills became dislodged after a few seconds (at ≈5 s), the ALG-DA pills remained adherent to the small intestine for more than 10 min, at which time the measurement was stopped (Figure 4C).

We measured the size of the pills with a caliper at predetermined time intervals during the water flushing experiment. ALG pills were used as controls, and graphite was added to the ALG pills to investigate the erosion process (dark color) (Figure 4D). With the increase of water flushing time from 0 to 30 min, the sizes of ALG-DA pills increased 1.2 times due to the forming of hydrogels. The sizes of ALG pills decreased due to ALG dissolved and eroded from pills. After 30 min of flushing, ALG pills were eroded completely, while ALG-DA pills remained in place (Figure 4E). These results further confirmed that the ALG-DA pills showed the significantly longer duration than ALG pills.

The mucoadhesion of the ALG-DA hydrogel can be further increased in the presence of oxidizing agents during contact with mucin. However, if the gel is fully oxidized before contacting mucin, the mucoadhesion enhancement will become weak.^[51]^ This was confirmed by the observation that ALG-DA pills remained adherent to the intestinal surface for >10 min in the water flushing experiment, while pills made from alginatepolydopamine dislodged in this period. The results were shown in Figure S5 and Movie S3 (Supporting Information).

### 2.5. In Vitro Release

**Figure 5.**
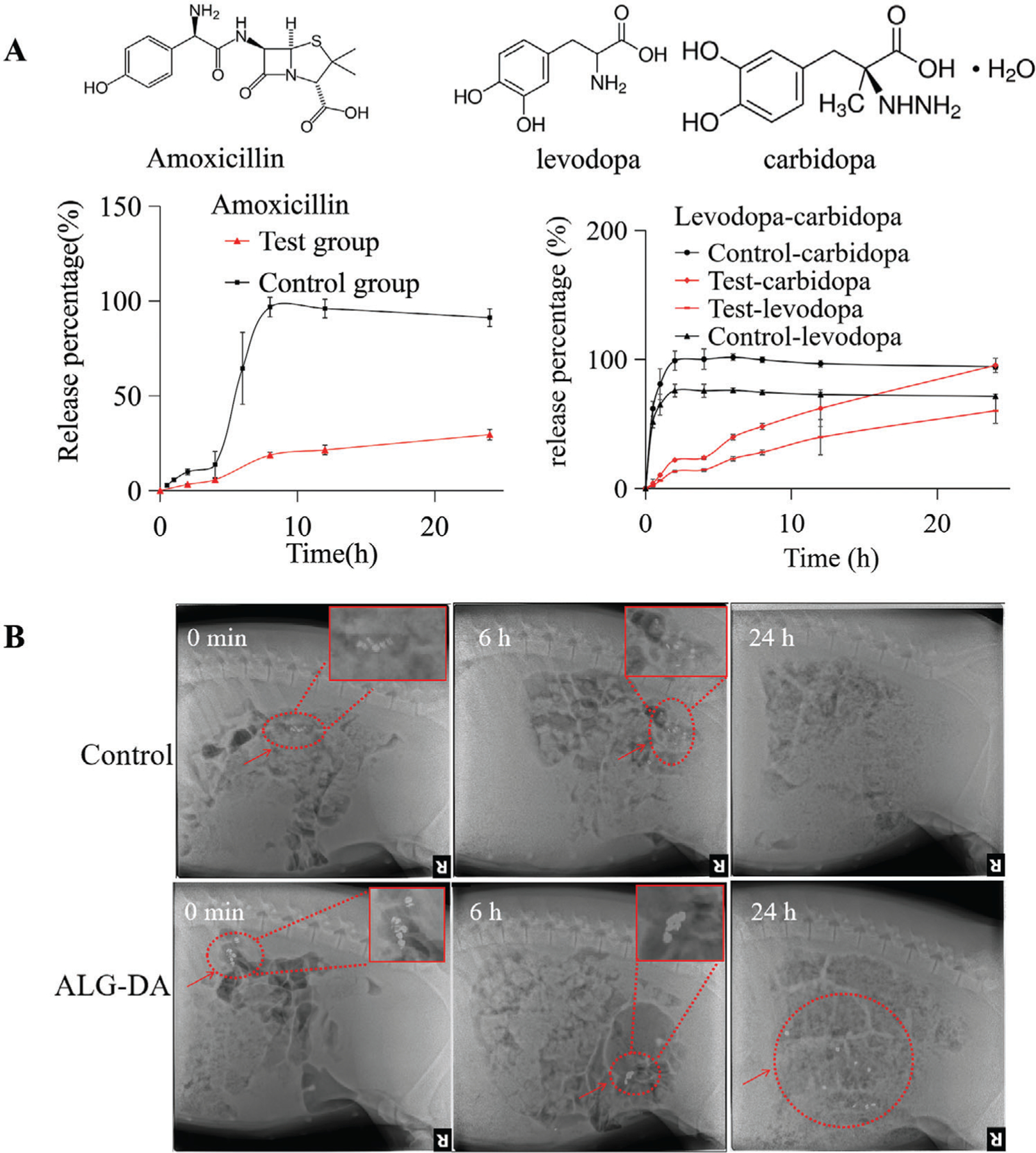
Drug delivery evaluation in vitro and in vivo. A) Mean in vitro drug release of amoxicillin and levodopa/carbidopa in middle layer of triple-layer pills (*n* = 3). B) GI tract residence of control pills and alginate-dopamine (ALG-DA) pills via X-ray imaging. Data is represented as mean ± S.D., *n* = 3.

We fabricated the middle-layer of pills with hydroxypropyl methyl cellulose (HPMC) and loaded it with a hydrophobic drug combination (levodopa and carbidopa) or a hydrophilic drug (amoxicillin) (Figure 5A). HPMC was selected as a drug release matrix because of its hydrophilic nature and capacity to produce sustained drug release. We prepared various drug formulations by mixing drugs and HPMC in different ratios (Figure S6, Supporting Information).

We assembled drug-HPMC pills for the hydrophilic antibiotic amoxicillin, which is used for the treatment of pediatric pneumonia, and for the hydrophobic levodopa/carbidopa combination, an anti-Parkinson’s disease drug combination. As compared with the control group (lactose pills), the drug release rate from the HPMC matrix was sustained for amoxicillin, levodopa, and carbidopa. Furthermore, the HPMC formulations (test group) showed a linear kinetic profile with limited burst release of amoxicillin and levodopa/carbidopa (Figure 5A). We tested the influence of HPMC percentage on drugs’ release profiles. Drug release from pills was studied in PBS for amoxicillin and PBS containing 2% sodium dodecyl sulfate for the levodopa/carbidopa (see Figure S6, Supporting Information). A range of HPMC matrices were tested for drug release: one containing 10% HPMC (low viscosity), one containing 20% HPMC (low viscosity), the third containing 50% HPMC (high viscosity). The rate of release of amoxicillin from 50% HPMC (high viscosity) was slower than other three matrices, and all formulations showed a linear release kinetic profile. We chose 50% HPMC (high viscosity) in order to support prolonged release times during the retention in the small intestine. For levodopa/carbidopa, the release from pills with 20% HPMC (low viscosity) showed more sustained and linear profile than 10% HPMC (low viscosity). Hence, we chose 20% HPMC (low viscosity) as the lead formulation for our follow-up studies.

### 2.6. In Vivo Retention Evaluation

We characterized the intestinal residence of our dosage forms in female Yorkshire pigs (50 to 80 kg) from Tufts University (Grafton, MA) using periodic radiographs. Small intestinal residence was evaluated by periodic radiographic evaluation. Under anesthesia, pills were introduced using an overtube and endoscope into the small intestine. Representative serial abdominal radiographs after administration revealed that the ALG-DA triple-layer pills remained in the intestinal tract for over 24 h. For the control pills (lactose pills), there were no radiographic signals at G h (Figure 5B).

### 2.7. Pharmacokinetics in a Large Animal Model

**Figure 6.**
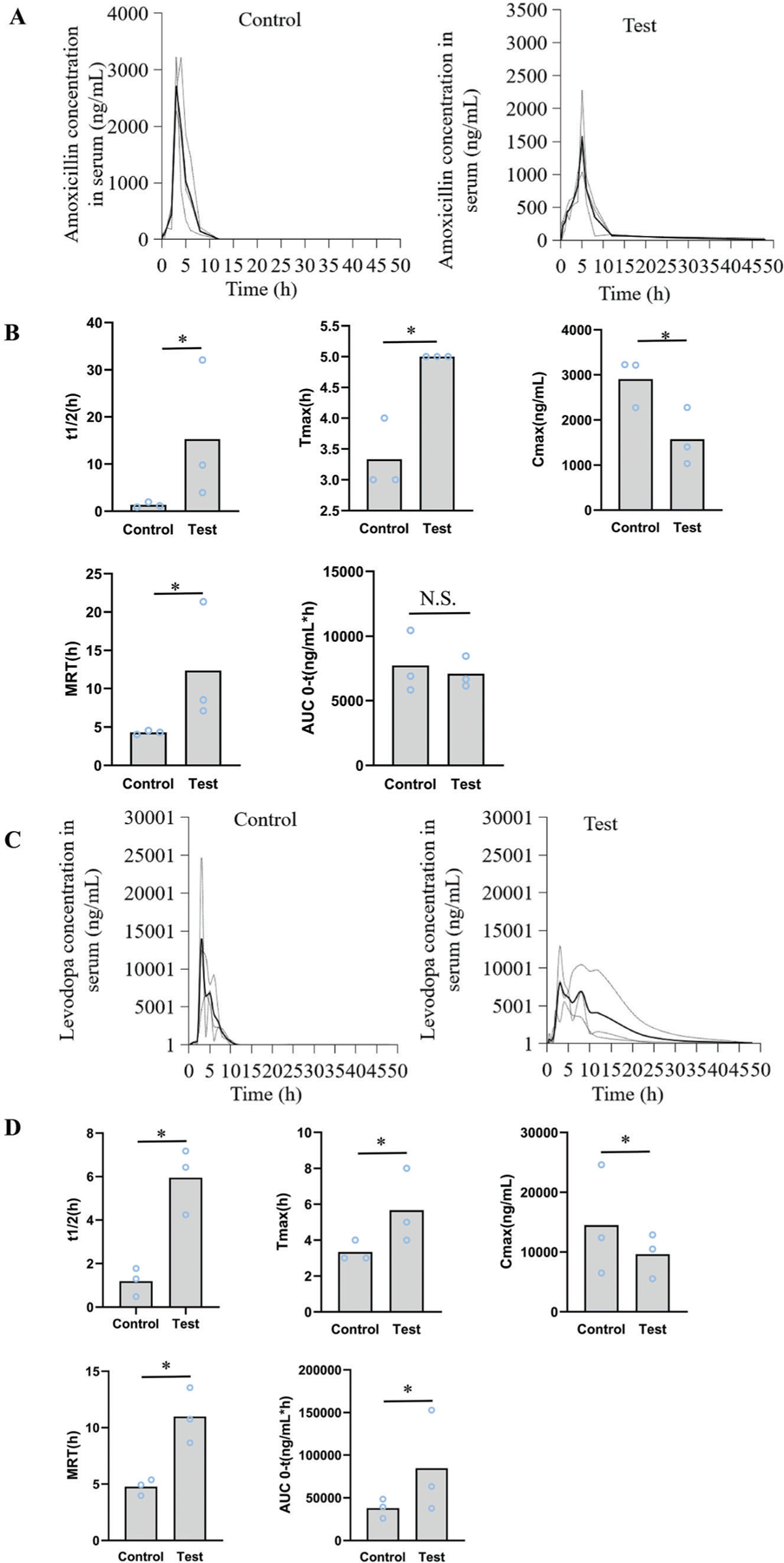
Drug delivery pharmacokinetic evaluation in vivo. A) The serum concentration-time curve of amoxicillin when administered as control pills and ALG-DA pills in pigs. Data is represented as mean ± S.D., *n* = 3. B) The pharmacokinetic parameters of amoxicillin when administered as control pills and ALG-DA pills in pigs. Data is represented as mean ± S.D., *n* = 3. C) The serum concentration-time curve of levodopa when administered as control pills and ALG-DA pills in pigs. Data is represented as mean ± S.D., *n* = 3. D) The pharmacokinetic parameters of levodopa when administered as control pills and ALG-DA pills in pigs. Data is represented as mean ± S.D., *n* = 3. *indicates *p* < 0.05, which demonstrates that differences are statistically significant. N.S. indicates that differences are not statistically significant, independent sample T-test.

The ALG-DA triple-layer pills containing amoxicillin in the middle layer were administered (ALG-DA pills, test group) in the intestine of female Yorkshire pigs (50 to 80 kg) from Tufts University (Grafton, MA) under anesthesia. Pills containing amoxicillin with lactose were used as the control (Control pills, control group). The pharmacokinetic parameters (including half-life (t1/2), mean retention time (MRT), maximum concentration (Cmax), peak time (Tmax), and area under the curve (AUC)) of drugs in pigs were calculated using a noncompartment pharmacokinetics model. Results demonstrated that a 11.5-fold increase between the t1/2 of amoxicillin test group and control group. Tmax increased by 1.5-fold (from 3.3 to 5.0 h), MRT increased by 2.9-fold (from 4.3 to 12.3 h) (*p* < 0.05, compared with control group,

Independent Samples T-Test, SPSS), which demonstrated that our developed universal pill formulations using the GSEL technology prolonged intestinal residence of hydrophilic drug-amoxicillin. There is no significant difference between the AUC of amoxicillin test group and control group (*p* > 0.05, compared with control group,

Independent Samples T-Test, SPSS) (Figure 6A,B). The PK parameters are shown in Table 1.

We then analyzed the pharmacokinetics (PK) of the levodopa-carbidopa administered as immediate-release formulations (Control pills with lactose containing both levodopa and carbidopa, control group) and our ALG-DA triple-layer pills (ALG-DA pills containing both levodopa and carbidopa, test group). Results demonstrated that a 5-fold increase between the t1/2 of levodopa test group and control group. Tmax increased by 1.7-fold (from 3.3 to 5.7 h), MRT increased by 2.3-fold (from 4.7 to 11 h) (*p* < 0.05, compared with control group, Independent Samples T-Test, SPSS), which demonstrated that our developed triple-layered pill formulation using the GSEL technology prolonged intestinal residence of hydrophobic drug-levodopa. Importantly, a prolonged half-life can result in sustained clinical benefit. AUC of levodopa in test group increased 2.2-fold compared with control group (*p* < 0.05, compared with control group, Independent Samples T-Test, SPSS) (Figure 6C,D). Cmax after a single-dose administration of ALG-DA pills decreased. Reduced peak plasma concentration may decrease the symptoms of high concentration (peak level), such as hand tapping, walking time.^[52]^ The PK parameters are shown in Table 1.

### 2.8. Safety Evaluation In Vitro, Ex Vivo, and In Vivo

In order to translate mussel-inspired wet adhesive into clinical practice, its biocompatibility is of paramount interest.^[53]^ Hence, we evaluated the biocompatibility of the ALG-DA hydrogels in in vitro and in vivo studies.

We first assessed the safety of the material by testing its cytotoxicity in vitro.^[54]^ HEK293T cells, Caco-2 cells, Hs888Lu cells, HT29 cells and HepG2-C3A cells were used to assess cytotoxicity of ALG-DA hydrogel. The cells were treated with extracts of ALG-DA hydrogel and cell viability was measured using 3-(4, 5-dimethylthiazol-2-yl)−2,5-diphenyltetrazolium bromide (MTT) test. The relative cell viabilities were all above 80% for all groups after G and 24 h incubation (Figure S7A,B, Supporting Information).

For in vivo safety evaluation in rats, 2 groups of rats were used with 5 rats in each group. One group was administered water (control group). Another group was administered ALG-DA based pills powder, including ALG-DA, DA, H_2_O_2_, Tris (test group). We gavaged rats every day for 1 month and weighed them every day (Figure S8, Supporting Information). There were no changes in behavior and no treatment-related morbidity or mortality was observed. Very mild weight loss was observed which is likely due to the barrier function from the combined ALG-DA system which had been suggested by the transient barrier formation by GSEL nutrient absorption we observed in our prior study. The blood was taken for biochemical analysis (Table S1, Supporting Information) and complete blood counts (Table S2, Supporting Information), which demonstrated that there was no abnormity compared with the reference ranges. We then harvested the brain, heart, liver, spleen, lung, kidney to perform histological analysis. The results were listed in Figure S9a–i (Supporting Information), which demonstrated that there were no adverse reaction.

We also tested local toxicity of the ALG-DA pills in swine tissue ex vivo. Pills were fixed on the small intestine of pigs, and after G h, the tissue attached to the pills was harvested and processed for histology. Notably, small intestinal mucosal surfaces did not show any injuries (Figure S9j, Supporting Information).

## 3. Discussion

In this study we describe in situ formation of adhesive material for oral drug delivery. Sodium alginate was conjugated with dopamine. In situ oxidation of catechol groups in dopamine to quinones leads to covalent cross-linking and adhesion to biological substrates.^[55]^ Moreover, quinone can also react with various nucleophilic functional groups (i.e., –NH2, –SH, imidazole) found on biological substrates, forming interfacial covalent bonds. The content of catechol is the key to maintain a balance between hydrophobic and electrostatic interactions for tuning or optimizing both coacervation and adhesion. Despite a decade of development, mussel inspired adhesives applied to the oral drug delivery have yet to be clinically translated, due to some barriers, particularly with respect to practical load-bearing applications. For example, the stomach seems to have a limited capacity of absorption and may decarboxylate levodopa, moreover, large intestinal microbiota can dehydroxylate levodopa to dopamine,^[56]^ which increases the dosage regimen of levodopa treatment in PD. It has a pivotal role in regulating the delivery of the ingested levodopa dose to the proximal small bowel, where most of the levodopa absorption takes place.^[57]^ Using in situ intestinal enzymeresponsive components with an ultrafast and robust response enables the development of a new generation of GI bio-adhesive platform.

Antibiotics are used to treat a wide variety of infections and decreasing the frequency of administration stands to have a positive impact on adherence. The case similar for treatments for Parkinson’s disease where treatments are essential in supporting and maximizing neurologic motor function. Here, we report the design and preliminary testing of an oral dosage form that specifically adheres to the small intestine. To develop this platform, several barriers needed to be overcome. First, large drug loading may influence the adhesion of our polymer. Second, the other excipients’ contents may influence the gelling homogenization. To address the dose loading challenge we designed a triple layered pill where the middle layer is able to load and release various drugs and the upper and lower layers are mixed with ingredients that can react homogeneously to support mucosal adhesion. However, hydrogels can be fragile, which may limit their use in most load-bearing applications. To ensure the systems could be stored under dry conditions, all components were blended in a dry powder form, which could then interact, gel, and form the final adhesive when in contact with moist tissue, in case of rapid gelation leading to inhomogeneous mixing and poor performance. For the middle layer, drugs are encapsulated in polymer matrices. These formulations released drug with a profile with near zero-order release kinetics.

We tested the triple layered system with the release of amoxicillin and levodopa by delivering them to the small intestine. We used a swine model for studying the intestinal residence and the drugs’ PK, as the anatomy of the pig intestine closely resembles that of humans’, and pigs used in this study had a body weight comparable to adult humans. The control group produced a peak concentration after which the concentration declined throughout the study. In contrast, the ALG-DA group had a longer t_1/2_ than control group. For levodopa/carbidopa, they’re hydrophobic drugs and have good intestinal permeation. Through increasing the Tmax or MRT using our developed hydrogel system, we can improve their AUC_0-t_. For Amoxicillin, AUC_0-t_ is not improved significantly, because it’s hydrophilic drug, our hydrogel system can just prolong it’s Tmax or MRT and can’t enhance it’s intestinal permeation. We can add some permeation enhancers to improve AUC_0-t_ using our hydrogel system in the future.

For in vivo evaluation of retention or PK, we delivered pills via an overtube and with endoscopic assistance to the small intestine of pigs to investigate the ALG-DA pills’ properties. Future successful human translation will require further safety evaluation in human and optimal in vivo pill delivery devices to the small intestine, such as intestinal pill release capsules similar to our group’s LUMI, “jack-in-the-box”system and DOAMS,^[43,58]^ which can eject our pills directly into the intestinal cavity, inducing appropriate contact with specialized pills.

## 4. Conclusion

Dopamine in alginate-dopamine is localized at the surface to provide adhesion, while the alginate is cross-linked to provide bulk cohesion. PDA also links everything together, and the components diffuse and harden to form the final hydrogel. We showed here that the development of mucoadhesive triple-layer pills can enable both hydrophilic and hydrophobic in both pediatric and geriatric drugs’ oral sustained delivery. With careful optimization, pills can load a range of drugs with high doses, while avoiding interference with the mussel inspired adhesive’s mechanical and gelling properties, which prolongs the t_1/2_ of drugs and reduces the dose frequency. We reasoned that a technology that reduces dosing frequency and is administered orally could be a promising solution for addressing imperfect adherence to chronic disease. Given the in situ triggered properties of the hydrogel, we anticipate that our intestinal enzyme-triggered mucoadhesive system could usher in more development of drug delivery, biomedical application and sensing systems in the GI tract.

## 5. Experimental Section

### Materials:

Alginic acid sodium salt from brown algae, Dopamine hydrochloride, barium sulfate, dimethylsulfoxide, Trizma base, Phosphate-buffered saline (PBS, pH 7.4), Hydrogen Peroxide, (Hydroxypropyl)methyl cellulose were available commercially from Sigma-Aldrich. EDC (1-ethyl-3-(3-dimethylaminopropyl)carbodiimide hydrochloride), N-Hydroxysuccinimide, BupH^™^ MES Buffered Saline, Sodium Dodecyl Sulfate (SDS), Deuterium oxide, Hydrochloric Acid Solution, Graphite (ACROS Organics^™^), Levodopa(USP, 98–102%, Spectrum^™^), Carbidopa (USP, 98–102%, Spectrum), L-(−)-𝛼-Methyldopa (hydrate) (98%), Phosphate-Citrate Buffer(Ph5.5), Vybrant^™^ MTT Cell Viability Assay, Hydroxypropyl methylcellulose were purchased from Fisher scientific. Nanopure water (18 MΩ cm) was acquired by means of a Milli-Q water filtration system, Millipore (St. Charles).

### Synthesis of Alginate-Dopamine:

Alginate modified with dopamine was synthesized using EDC-NHS chemistry. In brief, alginate (500 mg) was dissolved in MES (PH 4.7, 50 mL) solution. The solution was mixed with EDC and NHS at a 1:2:2 molar ratio of alginate monomer, EDC and NHS and reacted for 1 h. Then dopamine was added into the reaction solution at a 2:1 or 1:1 molar ratio to alginate monomer and reacted for over 12 h. The pH was maintained at 5.0. The reaction mixture was dialyzed using PBS (Adjusting pH 5.5 using 1N hydrochloric acid solution) and distilled water (Adjusting pH 5.5 using 1N hydrochloric acid solution) for 12 h. The resulting conjugate was lyophilized for 5 days and then stored at −20 °C.

### Ultraviolet–Visible Spectrophotometry:

Ultraviolet–visible spectrophotometer (Bruker, Billerica, MA, USA) was used to confirm the introduction of dopamine on the alginate backbone. A solution of ALG-DA (1 mg mL^−1^) in phosphate-citrate buffer at pH 5.5 was prepared and placed in 1 cm quartz cells. The wavelength used for this analysis was 280 nm. Phosphatecitrate Buffer (pH 5.5) was used as the reference solution.

### Nuclear Magnetic Resonance:

1H-NMR analyses were carried out by dissolving the alginate, dopamine and alginate-dopamine in deuterated water (D_2_O) (The concentration is 1 mg mL^−1^). The spectra were obtained using a spectrometer Bio Spin 400 MHz (Bruker, Billerica, MA, USA). The spectra were recorded at 298 K and 400 MHz for 1 H.

### Infrared Spectroscopy:

Infrared spectra were recorded on a FTIR6700 Fourier Transform Infrared Spectrometer (Bruker, Billerica, MA, USA) and analyzed using OPUS v. 6,5,92 software.

### Scanning Electron Microscope:

Surface morphology of the dehydrated alginate-dopamine hydrogels were observed using the scanning electron microscope FlexSEM 1000 II. 2% (W/V) and 4% (W/V) hydrogels were lyophilized and fixed to aluminum stubs with double-sided adhesive carbon conductive tape and subsequently sputter coated with carbon using a SC 7620 mini sputter coater, then scanned at 5.0 kV voltages and 50 Pa vacuum conditions with ultra-variable-pressure detector (UVD), respectively.

### Rheological Properties Test:

Rheological properties of the ALG-DA hydrogels were characterized using a TA Instruments DHR-2 Rheometer. ALG-DA dissolved in PBS (at 2% and 4% (w/v) concentration) was mixed with tris, H_2_O_2_ and catalase to initiate cross-linking of ALG-DA. The moduli of the hydrogels were measured using an angular frequency (0.1–100 rad s^−1^ at 1% strain) sweep. Hydrogel discs (diameter = 8 mm, *n* = 3) were tested using parallel plates at a loading gap distance that was set at 5000 μm that of the individual hydrogel thickness, as measured by a digital caliber.

### Swelling of Alginate-Dopamine:

The swelling of ALG-DA hydrogels was measured by incubating the samples in PBS at 37 °C on a shaker at 100 rpm and subsequent measuring the weights at predetermined time intervals, then comparing with their initial weights.

### Gelation In Vitro and In Vivo:

All tissue and in vivo animal experiments were performed in accordance with the approval of the MIT Committee on Animal Care (approval no. 0919–058-22). The bio-adhesive layers of triple-layer pills was prepared and tested their gelation in vitro and in vivo. The covalent reaction of dopamine-based polymer changed color, the hydrogel becomes dark, which was also the sign of gelation. Pills’ gelation was tested on different portions of porcine GI tract (esophagus, stomach, small intestine, colon) at different time-points. Pictures were taken of the pills and their darkness were calculated via image J software to test the gelation degree. ImageJ/FIJI – measure tool was used to quantify the color change of the pill over time. Images were converted to grayscale before processing. For processing, a rectangular contour of 1 mm^2^ was used as a selection mask to repeatedly measure the intensity using Analyze tool. Each pill signal intensity was determined by the relative intensity ratio compared to the small intestine as a background. Five measurements were taken for each pill across the pill area. The equation: Darkness ratio (%) = (Brightness intensity value of the small intestine-brightness intensity value of the pill)/Brightness intensity value of the small intestine×100%. The pills were delivered to the intestine of pigs through an over-tube, and then took pictures to investigate the gelation of pills on the small intestine of pigs in vivo.

### Adhesion Test In Vitro:

Adhesion studies were performed by employing an experimental setup (Figure 4A). Excised porcine intestinal tissues were cut and opened to line the slide of the apparatus. With the detachable slide from the apparatus laid flat, alginate-calcium pills (ALG-Ca) and alginate-dopamine pills (ALG-DA) were placed on the tissue at a shape of MIT. They were incubated at room temperature for 10 min allowing the pills to get wet. The slide was turned upside down to ensure that the devices had adhered and was put back to the apparatus at a tilt angle of 45°. At room temperature, the fixed mucosal intestinal tissue was continuously flushed with water. The times for dislodgment were documented and compared for the different formulations. Videos were recorded with a digital camera and sequential photographs from the video recordings were collected. Oxidation products of ALG-DA pills (ALG-PDA) and ALG-DA pills were placed on the tissue, they were continuously flushed with water, the video and pictures were recorded with a digital camera at predetermined time intervals.

### Erosion Test In Vitro:

The erosion of alginate-dopamine pills (ALG-DA) was measured by placing them on the tissue, alginate pills (ALG) as a control. The apparatus was set at a tilt angle of 45°. At room temperature, the fixed mucosal intestinal tissue was continuously flushed with water. Pictures of pills were recorded with a digital camera and the sizes of pills were measured by a caliper at predetermined time intervals. Four replicates were conducted for each sample.

### In Vitro Drug Release:

The pill was submerged in PBS (50 mL) in a VWR (50 mL) centrifuge tube, three replicates for each time point and condition were incubated at 37 °C on a shaker plate at 100 rpm. At each time point, release medium (1 mL) was replaced by fresh medium (1 mL) and then kept in refrigerator until analysis. The release study was carried out for 24 h and the total drug release was measured by HPLC.

### In Vivo Residence Test:

All animal studies were approved by the Committee on Animal Care at the Massachusetts Institute of Technology (CAC Protocol Number:0919–058-22). To assess pills’ retention in small intestine cavity, radiopaque barium sulfate-labeled pills were administered via the over tube into the small intestine of female Yorkshire pigs (50 to 80 kg) from Tuft University (Grafton, MA). Pigs were anesthetized with an intra-muscular injection of Telazol (5 mg kg^−1^), xylazine (2 mg kg^−1^), and atropine (0.04 mg kg^−1^). They were incubated and maintained on isoflurane gas anesthesia. Pills were introduced using an overtube and endoscope into the small intestine. Radiographs were performed at different time-points to monitor the transit of the pills.

### Oral Pharmacokinetics Studies:

All procedures were conducted in accordance with protocols approved by the Committee on Animal Care at the Massachusetts Institute of Technology (CAC Protocol Number:0919–058-22). The pharmacokinetics (PK) of amoxicillin and levodopa/carbidopa administered was prepared as a standard-release pill (Control pill, Amoxicillin, 8 mg of drug per Kg pig, Levodopa, 10 mg of drug per Kg pig, Carbidopa, 2.5 mg of drug per Kg pig) or an ALG-DA triple-layer pill (ALG-DA pill, Amoxicillin, 20 mg of drug per Kg pig, Levodopa, 10 mg of drug per Kg pig, Carbidopa, 2.5 mg of drug per Kg pig) in female Yorkshire pigs (50 to 80 kg) from Tuft University (Grafton, MA). Pigs were fed daily in the morning and in the evening, with a diet consisting of pellets (Laboratory Mini-Pig Growler Diet, 5081), with a midday snack consisting of various fruits and vegetables. Pigs were on liquid diet for 24 h before the procedure and fasted overnight. Pigs were sedated with intramuscular injection of midazolam (0.25 mg kg^−1^) with dexmedatomidine (0.03 mg kg^−1^) (survival studies). For longer hour study or terminal days, pigs were anesthetized with an intramuscular injection of Telazol (5 mg kg^−1^), xylazine (2 mg kg^−1^), and atropine (0.04 mg kg^−1^). They were incubated and maintained on isoflurane gas anesthesia. An over-tube was placed with endoscopic guidance into the proximal intestine. The pills were placed into the intestine via the over-tube, one dosage form per animal. After treatment administration, the over-tube was carefully removed. At various times, blood was drawn from the mammary vein and transferred to a BD Vacutainer serum separator tubes (Becton, Dickinson and Co.). The tubes were centrifuged (3202 g, 10 min, 4 °C), and the serum was collected and stored at −80 °C until further analysis. The samples were analyzed by liquid chromatography–tandem mass spectroscopy (LC-MS/MS) for serum amoxicillin and levodopa concentration.

### Cytotoxicity Assay:

The alginate-dopamine hydrogel was incubated in the culture medium with a range of dosage from 0.2 to 2 mg mL^−1^ at 37 °C for 6 or 24 h. The obtained medium was then tested for its toxicity toward cells. Cell lines were purchased from ATCC for these experiments. Cytotoxicity was tested on HEK293T cells, Caco-2 cells, Hs888Lu cells, HT29 cells, and HepG2-C3A cells by seeding them each in a 96-well plate at a density of 10000 cells per well. Cells were kept in culture for 24 h before replacing the medium with the pre-prepared solutions (100 μL) as described above. After 6 or 24 h culture, these solutions were replaced with untreated media (100 μL) and cytotoxicity was quantified by adding MTT reagent (10 μL) to each well. The contents were mixed and then allowed to be incubated at 37 °C for 4 h. Absorbance wavelength was recorded on an Infinite^®^ M200Pro (Tecan) with excitation at 540 nm. Cells that were not subjected to hydrogel-treated media provided a control. Before and after treated with hydrogel, cells lines were taken pictures to investigate their status. Cell viability was calculated by the following equation: cell viability (%) = [Absorbance(sample)−Absorbance(blank)]/[Absorbance(control)–Absorbance(blank)] × 100%.

### In Vivo Safety Evaluation:

All animal procedures were conducted in accordance with protocols approved by the Committee on Animal Care at the Massachusetts Institute of Technology (CAC Protocol Number:0919–058-22). In order to test in vivo toxicity of the polymers, the minimum of 5 rats (Sprague Dawley, Male, 250 g, Charles River, MA, USA) were dosed for each of groups. Pills were first pulverized to allow administration via oral gavage. This powder was weighed and re-suspended in sufficient water to obtain an ingestible suspension with the highest amount of material. The alginate-dopamine (The maximum dose is 60 mg kg^−1^ rats) was administered daily. Suspensions (10 mL kg^−1^ body weight) were gavaged orally. Control rats were administered water (10 mL kg^−1^). The weight, behavioral changes, and overall health of the rats were monitored daily for 28 days. After 28 days, animals were euthanized by CO_2_ asphyxiation and necropsied. Blood of each rats was harvested in serum separator tubes and spun down and the serum was aliquoted in a screw cap tube and frozen at −20 °C until further chemistry indexes analysis and whole blood for complete blood counts test. Brain, heart, liver, spleen, lungs, kidney, stomach, small intestine, large intestine were extracted weighed and sectioned for histopathological examination. An independent pathologist examined all organs to detect any abnormalities in a blinded fashion.

### Statistical Analysis:

The measurements were presented as means ± SD. Data were analyzed by independent sample T-test analysis of variance using SPSS. Between-groups differences were considered significant when *P* was < 0.05.

## Supplementary Material

Supplemental Movie 2

Supplemental Movie 1

Supplemental Movie 3

Supporting Information

## Figures and Tables

**Table 1.** Parameters of drugs pharmacokinetics in pigs plasma.

PK Parameters	Amoxicillin		Levodopa	
	Control group	Test group	Control group	Test group
T/2 (h)	1.33 ± 0.56	15.28 ± 14.85*	1.19 ± 0.65	5.95 ± 1.52*
Tmax (h)	3.33 ± 0.58	5 ± 0*	3.33 ± 0.58	5.67 ± 2.08*
Cmax(ng/mL)	2905.13 ± 546.33	1573.6 ± 638.64*	14 495.33 ± 9244.42	9633.3 ± 3734.54*
MRT (h)	4.3 ± 0.26	12.33 ± 5.5*	4.76 ± 0.72	10.98 ± 2.46*
AUC (ng/mL*h)	7732.25 ± 2418.25	7092.57 ± 1209.64	37 878.32 ± 11 330.11	84 626.71 ± 60 445.21*

* *P* < 0.05, compared with control group, which demonstrates that differences are statistically significant, Independent sample T-test.

## Data Availability

Research data are not shared.
