# Supplementary material for: Enzyme-Triggered Intestine-Specific Targeting Adhesive Platform for Universal Oral Drug Delivery
Source: Adv Healthc Mater. Author manuscript; Available in PMC 2023 Nov 16. (PMC10653991; doi:10.1002/adhm.202301033)
Supplement: Supplementary file 1 [file NIHMS1936962-supplement-Supporting_Information.pdf]

ADVANCED HEALTHCARE MATERIALS

Supporting Information

for *Adv. Healthcare Mater.*, DOI 10.1002/adhm.202301033

Enzyme-Triggered Intestine-Specific Targeting Adhesive Platform for Universal Oral Drug Delivery

*Ying Li, Jung Seung Lee, Ameya R. Kirtane, Mengyuan Li, Charles William Coffey III, Kaitlyn Hess, Aaron Lopes, Joy Collins, Siddartha Tamang, Keiko Ishida, Alison Hayward, Jacob Wainer, Adam J. Wentworth and Giovanni Traverso**

Supporting Information

Enzyme-triggered Intestine-specific targeting adhesive platform for universal oral drug delivery

*Ying Li[1,2], Jung Seung Lee[3,4], Ameya R. Kirtane[2,5], Mengyuan Li[2,6], Charles William Coffey III[7], Kaitlyn Hess[2], Aaron Lopes[2], Joy Collins[2], Siddartha Tamang[2], Keiko Ishida[2], Alison Hayward[2,8], Jacob Wainer[2], Adam J Wentworth[2,9], Giovanni Traverso[2,5,9]**

1 Institute of Medicinal Plant Development, Chinese Academy of Medical Sciences and Peking Union Medical College, Haidian District, Beijing 100193, P. R. China.

2 David H. Koch Institute for Integrative Cancer Research, Massachusetts Institute of Technology, Cambridge, MA 02139, USA.

3 Department of Intelligent Precision Healthcare Convergence, Sungkyunkwan University, Suwon, 16419, Korea

4 Department of Biomedical Engineering, Sungkyunkwan University, Suwon, 16419, Korea

5 Division of Gastroenterology, Brigham and Women's Hospital, Harvard Medical School, Boston, MA 02115, USA.

6 Faculty of Applied Science & Engineering, University of Toronto, Toronto, ON, M5S1A4, Canada.

7 Department of Biological Engineering, Massachusetts Institute of Technology, Cambridge, MA 02139, USA.

8 Division of Comparative Medicine, Massachusetts Institute of Technology, Cambridge, MA 02139, USA.

9 Department of Mechanical Engineering, Massachusetts Institute of Technology, Cambridge, MA 02139, USA.

* Corresponding author. Email: ctraverso@bwh.harvard.edu, cgt20@mit.edu (G.T.)

Methods

High performance liquid chromatography

High Performance Liquid Chromatography (HPLC) was used to determine the drug concentrations from all in-vitro release assays. An Agilent 1260 Infinity II HPLC system equipped with a quaternary pump, autosampler, thermostat, control module, and diode array detector. Data processing and analysis was performed using OpenLab CDS ChemStation®.

Levodopa and Carbidopa were separated on an Agilent Zorbax Eclipse XDB C18 analytical column 4.6 x 150 mm with 5 µm particles, maintained at 30 °C. The optimized mobile phase consisted of A: 10 mM sodium phosphate buffer at pH 3.00 and B: acetonitrile. Isocratic elution was employed over a 6 minute period to separate both compounds using a mobile phase composition of 90% A and 10% B. The injection volume was 5 µL, and the selected ultraviolet (UV) detection wavelength was 254 nm at a bandwidth of 4.0, no reference wavelength, and an acquisition rate of 10 Hz.

Amoxicillin was separated on a Phenomenex Kinetex PS C18 analytical column 4.6 x 150 mm with 2.6 µm particles, maintained at 30 °C. The optimized mobile phase consisted of A: 10 mM ammonium acetate at pH 4.00 and B: methanol. Isocratic elution was employed over a 5 minute period to separate both compounds using a mobile phase composition of 30% A and 70% B. The injection volume was 5 µL, and the selected ultraviolet (UV) detection wavelength was 230 nm at a bandwidth of 4.0, no reference wavelength, and an acquisition rate of 10 Hz.

Liquid chromatography tandem-mass spectrometry

Samples containing levodopa and carbidopa in swine serum from in vivo

experiments were prepared by protein precipitation and a simple derivatization protocol adapted from the method presented by Junnotula and Licea-Perez [1]. Stock solutions of each compound were prepared in methanol at a concentration of 500 µg/mL containing H3PO4 (1%v/v). A twelve-point calibration curve was prepared in black swine serum ranging from 10-25000 ng/mL. 100 µL of each sample was spiked with 50 µL of 500 ng/mL levodopa-d3 in acetonitrile as internal standards for levodopa. Then 400 µL of fluorescamine at 5mg/mL in acetonitrile was added to each sample. Samples were centrifuged for 10 minutes at 13,000 rpm. 300 µL was pipetted into fresh Eppendorf tubes and allowed to incubate covered at 37 °C for 60 minutes. Following incubation, 200 µL of supernatant was pipetted into a 96-well plate containing 200 µL of water. Analyte concentrations of levodopa and deuterated internal standard were analyzed using Ultra-Performance Liquid Chromatography-Tandem Mass Spectrometry (UPLC-MS/MS). Analysis was performed on a Waters ACQUITY UPLC®-I-Class System aligned with a Waters Xevo® TQ-S mass spectrometer (Waters Corporation, Milford MA). Liquid chromatographic separation was performed on either an Acquity UPLC® CSH (50mm × 2.1mm, 1.7 µm particle size) column at 50 °C. Sample introduction and ionization was by electrospray ionization (ESI) in the positive ionization mode. 10.0 µL was injected onto the instrument for analysis. For the analysis of levodopa and deuterated internal standards, the mobile phase consisted of aqueous 0.1% formic acid, 10mM ammonium formate solution (Mobile Phase A) and acetonitrile: 10 mM ammonium formate, 0.1% formic acid solution (95:5 v/v) (Mobile Phase B). The mobile phase had a continuous flow rate of 0.45 mL/min for using a time and solvent gradient composition. The initial composition, 95% Mobile Phase A, was held for 1.00 minutes. Following which, the composition was changed linearly to 5% Mobile Phase A and 95% Mobile Phase B until 1.25 minutes. The composition was held constant at 95% Mobile Phase B until 3.00 minutes. At 3.25 minutes the composition returned to 95% Mobile Phase A, where it remained for column equilibration for the duration of the run, ending at 4.00 minutes. Waters MassLynx 4.1 software was used for data acquisition and analysis. The mass to charge transition (m/z) used to quantitate levodopa fluorescamine was 458.16>139.137 and 461.16>232.4 for levodopa-d3 fluorescamine.

Swine serum sample preparation

Samples containing amoxicillin in swine serum from in vivo experiments were prepared for LC-MS/MS by protein precipitation. Stock solutions of amoxicillin and internal standard moxifloxacin were prepared in methanol at a concentration of 500 µg/mL. A twelve-point calibration curve was prepared in blank swine serum ranging from 2.5-10000 ng/mL. 100 µL of each sample was spiked with 200 µL of 250 ng/mL moxifloxacin in acetonitrile as the internal standard. Samples were centrifuged for 10 minutes at 13,000 rpm. Following centrifugation, 200 µL of supernatant was pipetted into a 96-well plate containing 200 µL of water. Analyte concentrations of amoxicillin were analyzed using Ultra-Performance Liquid Chromatography-Tandem Mass Spectrometry (UPLC-MS/MS). Analysis was performed on a Waters ACQUITY UPLC®-I-Class System aligned with a Waters Xevo® TQ-S mass spectrometer (Waters Corporation, Milford MA). Liquid chromatographic separation was performed

on either an Acquity UPLC® BEH (50mm × 2.1mm, 1.7 µm particle size) column at 50 °C. Sample introduction and ionization was by electrospray ionization (ESI) in the positive ionization mode. 10.0 µL was injected onto the instrument for analysis.For the analysis of amoxicillin, the mobile phase consisted of aqueous 0.1% formic acid, 10mM ammonium formate solution (Mobile Phase A) and acetonitrile: 10 mM ammonium formate, 0.1% formic acid solution (95:5 v/v) (Mobile Phase B). The mobile phase had a continuous flow rate of 0.6 mL/min using a time and solvent gradient composition. The initial composition, 100% Mobile Phase A, was held for 1.00 minutes. Following which, the composition was changed linearly to 50% Mobile Phase A and 50% Mobile Phase B until 1.25 minutes. At 1.50 minutes the composition changed to 20% Mobile Phase A, and at 2.50 minutes the composition was 100% Mobile Phase B. The composition was held constant at 100% Mobile Phase B until 3.00 minutes. At 3.25 minutes the composition returned to 100% Mobile Phase A, where it remained for column equilibration for the duration of the run, ending at 4.00 minutes. Waters MassLynx 4.1 software was used for data acquisition and analysis. The mass to charge transition (m/z) used to quantitate amoxicillin was 366.264>114.112 and 402.21>110.12 for internal standard moxifloxacin.

Figures and Tables

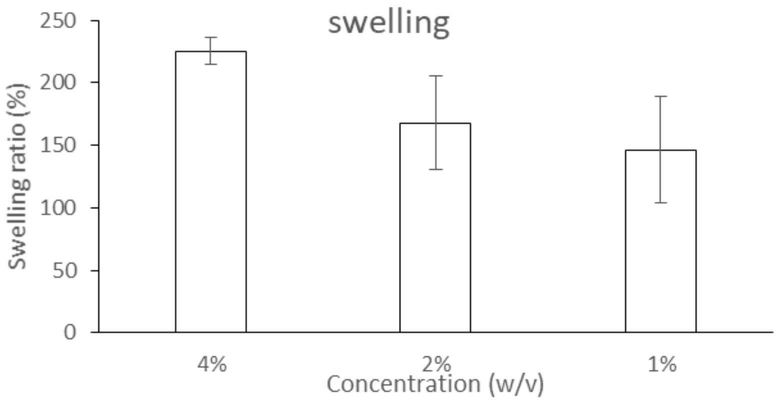

Figure S1. Swelling test of hydrogels. Swelling study performed to assess the cross-linking of 1 % (w/v)，2% (w/v)，4% (w/v) alginate-dopamine hydrogels. Data are reported as means ±S.D., n = 3.

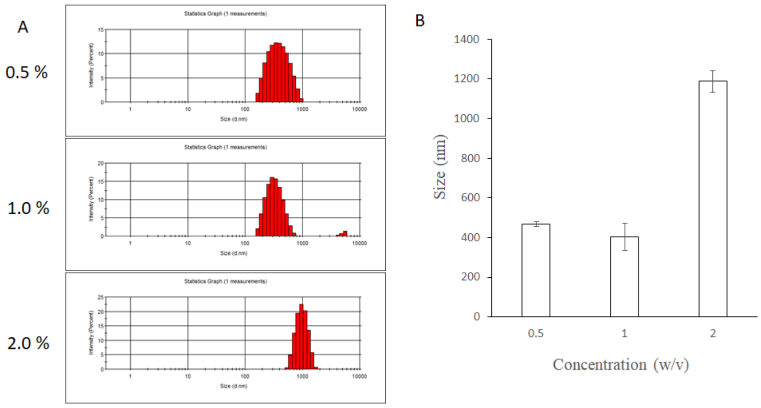

Figure S 2. Size test of polymerization of dopamine. (A) Graphs of polymerization of

dopamine (PDA) via dynamic light scattering (DLS). (B) Sizes of PDA for different concentration (0.5 %, 1 %, 2 % w/v) via DLS, Data are reported as means ±S.D., n=3.

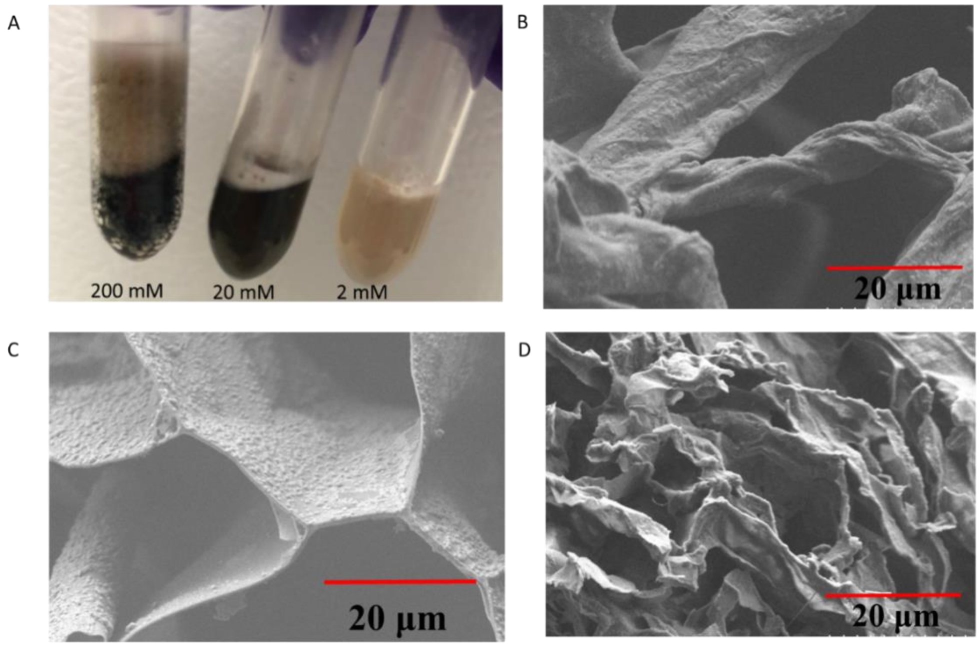

Figure S3. Images of hydrogels. (A) Photographs of Alginate-dopamine (2%W/V) with different concentrations of H2O2 within 3 minutes. (B) SEM image of Alginate-dopamine hydrogel (2%W/V) with 200 mM H2O2. (C) SEM image of Alginate-dopamine hydrogel (2%W/V) with 20 mM H2O2. (D) SEM image of Alginate-dopamine hydrogel (2%W/V) with 2 mM H2O2. Scale bar, 20 μm.

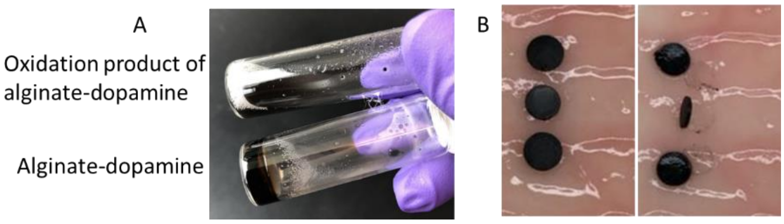

Figure S 4. Gelation test of oxidation products of alginate-dopamine. (A) The gelation of oxidation reaction products of alginate-dopamine (ALG-PDA) and alginate-dopamine in tris base buffer, H2O2 and catalase at a concentration of 2% (W/V). (B) The reaction of pills of ALG-PDA after 10 min attachment to the small intestine.

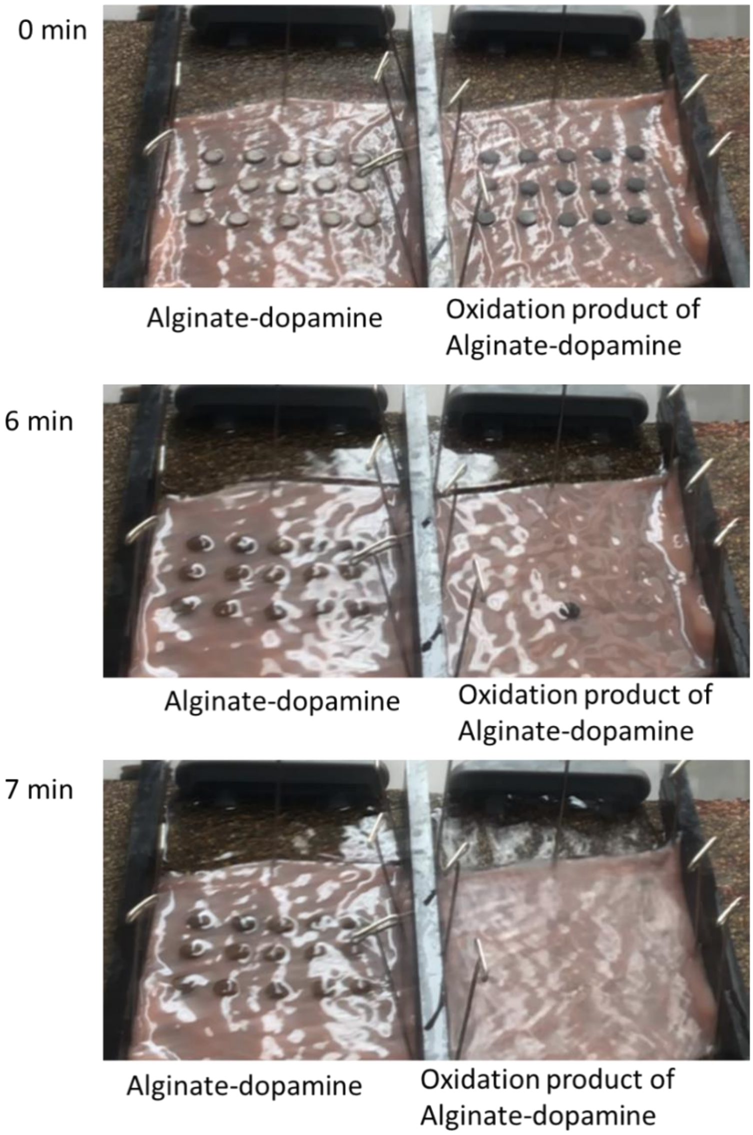

Figure S5. Adhesion test of oxidation product of alginate-dopamine. Digital photographs of adhesion on the small intestine of alginate-dopamine and oxidation product of alginate-dopamine using water flushing at different time-points.

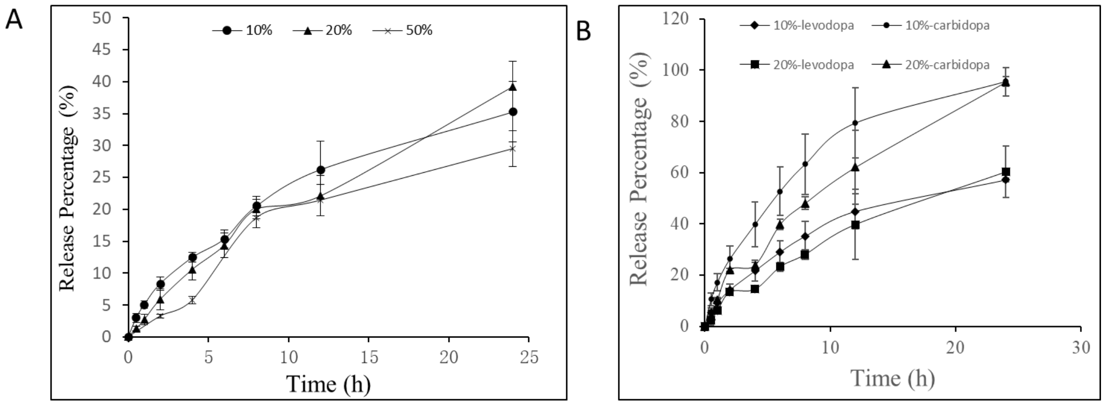

Figure S6. Drug release in vitro from pills of different formulations. (A) Amoxicillin release from pills composed of 10% HPMC (low viscosity), 20% HPMC (low viscosity)

and 50% HPMC (High viscosity). (B) Levodopa and carbidopa release from pills composed of 10% HPMC (low viscosity) and 20% HPMC (low viscosity). Data is represented as mean ± S.D., n = 3.

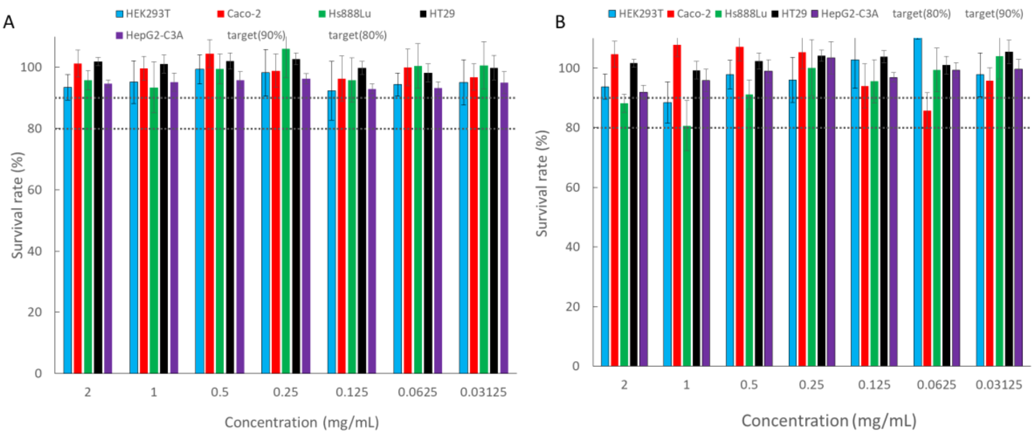

Figure S7. The biocompatible of alginate-dopamine hydrogel to various cell lines. (A) MTT assay of the proliferation of cells cultured with alginate-dopamine hydrogel for 6 hours (n =4). (B) MTT assay of the proliferation of cells cultured with alginate-dopamine hydrogel for 24 hours (n =4). Dotted lines indicate 80% and 90% survival rate.

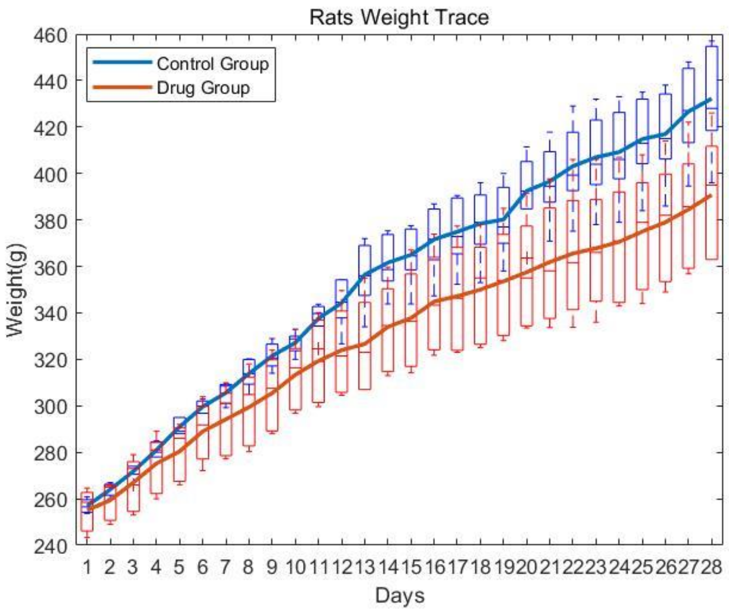

Figure S8. Weight of rats. Rats of control group were orally gavaged with water (n = 5) daily for 1 month, and rats of drug group were orally gavaged with pill formulation ingredients (60 mg per kg rat, n = 5) daily for 28 days.

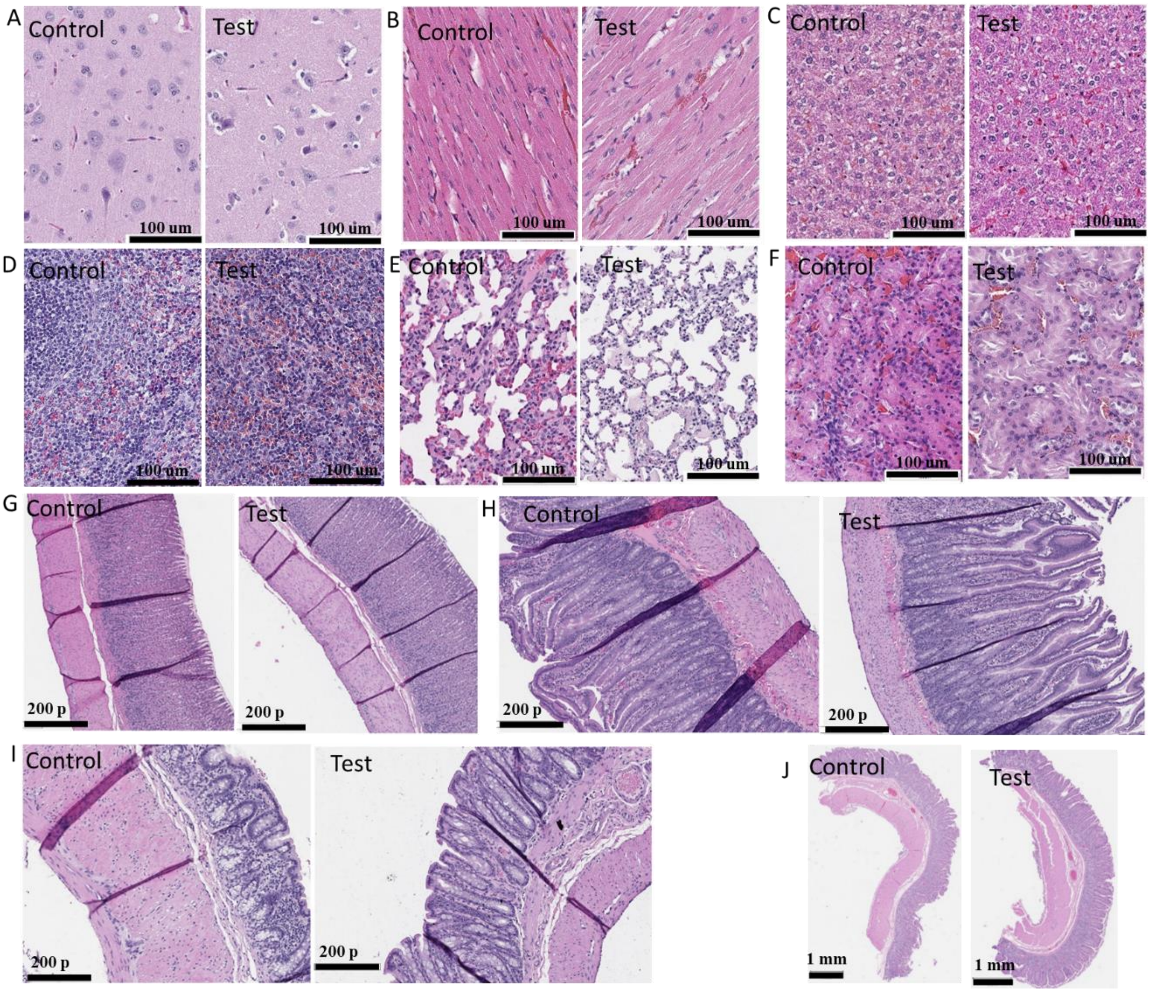

Figure S9. Representative histology images of rat tissues and pig small intestine. For the control group (left), SD rats were orally gavaged with the same volume of water (n = 5) daily for 28 days, and for the Test group (right), SD rats were orally gavaged with ALG-DA pill ingredients (60 mg per kg rat, n = 5) daily for 28 days.Scale bar, 100 μm or 200 p, (A) Representative histology images of rat brain, (B) Representative histology images of rat heart, (C) Representative histology images of rat liver, (D) Representative histology images of rat spleen, (E) Representative histology images of rat lung, (F) Representative histology images of rat kidney, (G) Representative histology images of rat stomach, (H) Representative histology images of rat small intestine, (I) Representative histology images of rat colon, (J) Representative histology images of pig small intestine. Pills were put on the small intestine for 6 hours via laparotomy. Scale bar, 1mm.

Table S 1. Biochemical indexes of rat blood after rats were gavaged with water (n = 5) and pill formulation ingredients daily for 28 days.

Biochemical indexes	Control group	Test group
ALK Phosphatase, IU/I	127.4 ±12.12	149.4 ±12.12
Gamma glutamyl transpeptidase (GGT), IU/I	0	0

AST (SGOT), IU/I	94.00± 8.76	93.2 ±12.0
ALT (SGPT)	41.8±5.5	42.4±5.5
TOTAL BILIRUBIN, mg/dl	0.14 ±0.055	0.10 ±0.00
Glucose	101.2±13.25	111±13.25
Urea Nitrogen, mg/dl	14.20 ±2.17	15.4 ±1.52
CREATININE, mg/dl	0.2 ±0	0.2 ±0
CHOLESTEROL, mg/dl	59.60 ±8.73	59.8 ±8.73
CALCIUM mg/dL	9.78 ±0.44	10.1 ±0.44
CHLORIDE, meq/l	99.5 ±1.73	100.5 ±1
PHOSPHORUS	9 ±0.81	9.76 ±0.81
POTASSIUM, meq/l	5.35±0.24	5.63±0.62
SODIUM, meq/l	142.25 ±0.96	142.75 ±1.5
A/G RATIO	1.26 ±0.089	1.24 ±0.089

Table S 2. Complete blood counts Test. Rats were gavaged with water (Control, n = 5) and pill ingredients (Test, n=5) daily for 28 days.

Biochemical indexes	Control group	Test group
White blood cells, K/µL	9.2 ±1.7	10.49 ±4.24
Neutrophils, K/µL	2.69±0.58	3.41±1.4
Lymphocytes, K/µL	5.9 ±1.4	6.27± 2.62
Monocytes, K/µL	0.59 ±0.15	0.79 ±0.42
Eosinophils, K/µL	0.025 ±0.03	0.028 ±0.021
Basophils, K/µL	0.0025±0.005	0±0
Red blood cells (M/µL)	7.72 ±0.48	8 ±0.6
Hemoglobin (g/dL)	16.3 ±0.84	16.7 ±0.38
Hematocrit (%)	47.3 ±2.72	50.78 ±3.65
Mean corpuscular volume (fL)	61.38±1.16	62.95±2.33
Mean corpuscular hemoglobin (pg)	21.13 ±0.52	20.78 ±0.95
MCHC (g/dL)	34.5 ±1.23	32.98 ±1.63
Red cell distribution width (%)	15.15±0.7	14.98±0.65
PLT (K/µL)	637.25±213.44	872.25±270.56
Mean platelet volume (fL)	6.53±0.26	6.25±0.4

Movie S1.mp4

Movie S1. Movie of adhesion test of alginate-calcium based pills.

Movie S2.mp4

Movie S2. Movie of adhesion test of alginate-dopamine based pills.

Movie S3.MOV

Movie S3. Movie of adhesion test of alginate-dopamine based pills and alginate-dopamine oxydate based pills.

References:

[1]V. Junnotula, H. Licea-Perez, *J. Chromatogr. B Analyt. Technol. Biomed. Life Sci.* **2013**, 926, 47-53.
